# RNA Splicing as a Therapeutic Target in Cancer

**DOI:** 10.1146/annurev-pharmtox-062124-035809

**Published:** 2025-07-22

**Authors:** Alexander M. Lewis, Kenyon Weis, Omar Abdel-Wahab

**Affiliations:** Molecular Pharmacology Program, Sloan Kettering Institute, Memorial Sloan Kettering Cancer Center, New York, NY, USA

**Keywords:** RNA, SF3B1, U2AF1, splicing, SRSF2, ZRSR2

## Abstract

RNA splicing is a nuclear enzymatic process that catalyzes excision of segments of premature messenger RNA (mRNA) and ligation to give rise to mature coding mRNA. Genomic and transcriptomic studies of cancer have revealed that RNA splicing is often dysregulated in cancer due to mutations in genes affecting their splicing in *cis*, alterations in the components of the splicing machinery in *trans*, and transcriptional as well as epigenetic alterations that impact cotranscriptional splicing. These observations have motivated a number of efforts to pharmacologically modulate splicing using small molecules that bind, degrade, or modify the RNA splicing machinery as well as oligonucleotides and small molecules that bind mRNA transcripts to modulate their processing. These therapeutic modalities are reviewed here along with early findings from clinical trials evaluating these agents in patients. The vast number of opportunities to alter splicing continues to highlight splicing as an exciting therapeutic target in cancer.

## INTRODUCTION

The discovery that protein-coding DNA is interspersed with non-protein-coding segments was one of the most surprising findings in early studies of the eukaryotic genome. Approximately 40 years ago, it was discovered that noncoding sequences (referred to as introns) are removed from premature messenger RNA (mRNA), and coding sequences (referred to as exons) are concatenated to generate mature mRNA via the process of RNA splicing. Splicing is catalyzed by a large macro-molecular machine composed of proteins and noncoding RNAs known as the spliceosome. RNA splicing occurs in the nucleus and is largely thought to occur in conjunction with transcription.

The process of RNA splicing affords the ability to generate multiple mRNA sequences from a single gene and give rise to potentially numerous distinct protein products. Moreover, by regulating the site of termination codons in an mRNA transcript, RNA splicing also regulates mRNA and protein stability. As such, cancer genomic studies over the last 15 years have revealed that RNA splicing is altered in cancer and, in turn, modifies gene expression as well as the repertoire of proteins produced in cancer cells. Given that RNA splicing is an enzymatic process that relies on numerous ATP-dependent catalytic steps, protein-protein interactions, protein-RNA interactions, and posttranslational modifications of splicing factors, numerous methods to modulate RNA splicing have been discovered. Although RNA splicing is an essential housekeeping function of eukaryotic cells, it is now clear that certain subsets of cancers are particularly sensitive to global modulation of splicing. Here we review existing and emerging approaches to modulate RNA splicing for therapeutic intent and highlight clinical results in early efforts to therapeutically alter splicing in patients with cancer (Supplemental Table 1).

## ALTERNATIVE SPLICING

The RNA splicing reaction is catalyzed by a macromolecular machine known as the major (or U2-dependent) spliceosome for 99.5% of human introns, while the remaining 0.5% of introns are spliced by the minor (or U12-dependent) spliceosome (1, 2). The assembly and mechanism of this complex oligomeric machinery have been described in detail by numerous previous reviews (2–9). One of the main advantages of the spliceosome is the generation of isoforms through a process known as alternative splicing (AS). AS is an essential mechanism for eukaryotic gene expression, allowing a single gene to produce multiple mRNA isoforms, significantly expanding proteomic diversity (3, 4). In addition, by defining the location of termination codons within a transcript, AS regulates stability and abundance of mRNA and thereby controls mRNA translation to protein.

The process of AS is governed by an interplay between *cis*-acting elements embedded in the mRNA and *trans*-acting factors that regulate splice site usage. *Cis*-acting elements include exonic splicing enhancers (ESEs) and silencers (ESSs), as well as intronic splicing enhancers (ISEs) and silencers (ISSs), which define exon inclusion or exclusion (4, 5). *Trans*-acting factors such as serine-arginine rich (SR) proteins and heterogeneous nuclear ribonucleoproteins (hnRNPs) recognize these *cis*-acting elements and recruit or inhibit spliceosomal components at specific splice sites (3, 10). This regulatory network is dynamic, with posttranslational modifications such as phosphorylation of SR proteins modulating their activity and subcellular localization (2, 3).

AS affects 95% of multiexonic human genes, contributing to tissue-specific gene expression, cellular differentiation, and developmental processes (1, 3, 6, 7). By selectively including or excluding exons, retaining introns, or even altering splice sites, AS generates isoforms with distinct and sometimes even opposing functions, enabling the fine-tuning of cellular responses (3, 7, 11). The dysregulation of splicing factors and regulatory networks of AS has profound implications in disease. Mutations in core splicing components, such as splicing factor 3b subunit 1 (SF3B1), serine- and arginine-rich splicing factor 2 (SRSF2), and U2 small nuclear RNA auxiliary factor 1 (U2AF1), disrupt splicing fidelity, leading to aberrant isoform production in cancers and hematological disorders (1, 4, 8, 11, 12). Aberrantly increased levels of AS and overexpression of oncogenic splicing factors, such as serine- and arginine-rich splicing factor 1 (SRSF1), promote the production of isoforms that enhance tumor survival and metastasis, while the loss of tumor-suppressive splicing factors exacerbates disease progression (9). The increasing development of technologies such as long-read single-cell RNA sequencing and advancements in therapeutic approaches targeting splicing regulation hold promise for detecting and restoring normal splicing patterns and mitigating pathogenesis (2, 3, 5, 8).

## MUTATIONS IN SPLICING FACTORS IN CANCER

Mutations in core-regulatory sequences of precursor messenger RNA (premRNA) can impair proper spliceosome recognition of an intron’s boundaries, branch point sequence, or polypyrimidine tract, contributing to improper intron/exon demarcation and subsequent aberrant splicing, thus driving cancer pathogenesis (13). Tumor progression can also be driven by impaired function of ESEs and ESSs, as well as ISEs and ISSs recognized by RNA-binding proteins that regulate splicing (14–16). Alternatively spliced transcripts in cancer have an elevated frequency of stop codons, and such alternatively spliced transcripts are found in greater amounts in tumors compared to normal tissue (17). Normal spliceosome processing can be impaired by disease-associated single-nucleotide polymorphisms and somatic missense mutations at critical splicing regulatory sites (18, 19). Aberrant RNA splicing derived from mutations in splicing factors is widespread in liquid and solid tumors, with the highest prevalence of mutations being found in acute myeloid leukemia (AML), chronic myelomonocytic leukemia (CMML), myelodysplastic syndromes (MDS), uveal melanoma (UVM), and chronic lymphocytic leukemia (CLL) (20).

The most common recurring splicing factor mutations in cancer affect the genes encoding SF3B1, SRSF2, U2AF1, and zinc finger CCCH-type, RNA-binding motif and serine/arginine-rich 2 (ZRSR2) and are present as heterozygous mutually exclusive mutations. Coexpression of these mutations in cells leads to intolerable cellular toxicity (3, 11, 21–25). Mutations in *SF3B1* are the most common RNA splicing factors across all cancers, with the highest prevalence in MDS, CLL, and UVM (26, 27). In MDS with refractory anemia and ringed sideroblasts, the prevalence of SF3B1 mutations is over 75% (26). The most common SF3B1 mutations affect the K700, R625, and K666 amino acid residues located in the C-terminal HEAT repeats of the SF3B1 protein, leading to cryptic splice sites and aberrant branch point selection (28, 29). It has been shown previously that G-patch domain containing 8 (GPATCH8) is a protein required for aberrant SF3B1 mis-splicing, and the therapeutic implications of correcting this mis-splicing have been demonstrated through silencing of GPATCH8, leading to improvements in mutant SF3B1 impaired hematopoiesis (28).

The second most mutated splicing factor in hematologic malignancies is SRSF2, which is present in approximately 50% of CMML cases, 20–30% of MDS, and 10–14% of AML (11, 30). SRSF2 mutations (P95) have been associated with worse overall survival, adverse prognoses, and a greater risk of transformation into AML (31, 32). SRSF2 encodes an RNA-binding protein and splicing factor that binds to ESEs with G- and C-rich sequences in its wild-type state (33, 34). SRSF2 mutations alter its RNA-binding specificity to promote the inclusion of mRNAs with C-rich ESEs. One key example of this aberrant RNA-binding and splicing activity is exemplified by SRSF2 mutant changes in enhancer of zeste homolog 2 (EZH2) mRNA where mutant SRSF2 promotes inclusion ofa poison cassette exon in EZH2, leading to the EZH2 transcript undergoing nonsense-mediated mRNA decay (NMD) and contributing to myeloid malignancy development (35, 36).

U2AF1 mutations are found in MDS, AML, and non-small-cell lung adenocarcinoma and are associated with high-risk MDS and adverse-risk AML (36–39). U2AF1 is a core spliceosomal component that binds to the AG dinucleotide that demarcates the 3′ splice site. U2AF1 mutations occur in its first (serine 34) or second (glutamine 157) zinc finger domain and impair recognition of the AG dinucleotide consensus sequence at the 3′ splice site and intron-exon boundary to alter 3′ splice site and cassette exon usage (40–43). Mis-splicing induced by U2AF1 mutations disrupts expression of genes encoding proteins in vital pathways, including apoptosis, DNA damage response, and DNA methylation (44).

ZRSR2 is a component of the minor spliceosome and helps with 3′ splice site recognition. Mutations in ZRSR2 lead to impaired minor intron removal (45). The U12 intron retention in ZRSR2 mutant cells generally leads to NMD of the aberrantly spliced transcript. Consistent with ZRSR2’s location on the X chromosome, mutations in ZRSR2 predominantly affect males with MDS, CMML, and AML and can lead to impaired dendritic cell inflammatory signaling, interferon production, and apoptosis (46).

## SF3B MODULATORY COMPOUNDS

### Noncovalent SF3B Inhibitors

Previous efforts to therapeutically target the spliceosome to alter RNA splicing have included the development of compounds that directly bind to the SF3B constituent of the U2 small nuclear ribonucleoprotein (snRNP). These compounds, FR901463, FR901464, and FR901465 (derived from *Pseudomonas* sp. 2663), disrupt early spliceosome assembly and promote cell cycle arrest in G1, impairing the growth of different cancers in vivo and in vitro (47, 48). Spliceostatin A, a methylated derivative of FR901464, is similar mechanistically to FR901464 and limits U1 availability for splicing (49, 50). Herboxidienes are herbicidal substances derived from *Streptomyces* sp. A7847, and one of the six forms of herboxidiene isolated, GEX1A, has shown effective antitumor activity in vitro and in vivo (51). Enhanced enantioselective total synthesis of herboxidiene also led to the development of a synthetic analog, 6-norherboxidiene, that has in vitro antitumor efficacy and can induce AS in *MDM2* premRNA (52).

Meayamycin B is another synthesized analog of FR901464 that has shown therapeutic promise when used in combination with ABT-737, an antagonist of BCL-2, BCL-XL, and BCL-w, effectively killing ABT-737-resistant non-small-cell lung cancer (NSCLC) cells in vitro (53, 54). Additionally, sudemycins have shown potential in treating mutant U2AF1 cancer cells (55).

**Figure 1:**
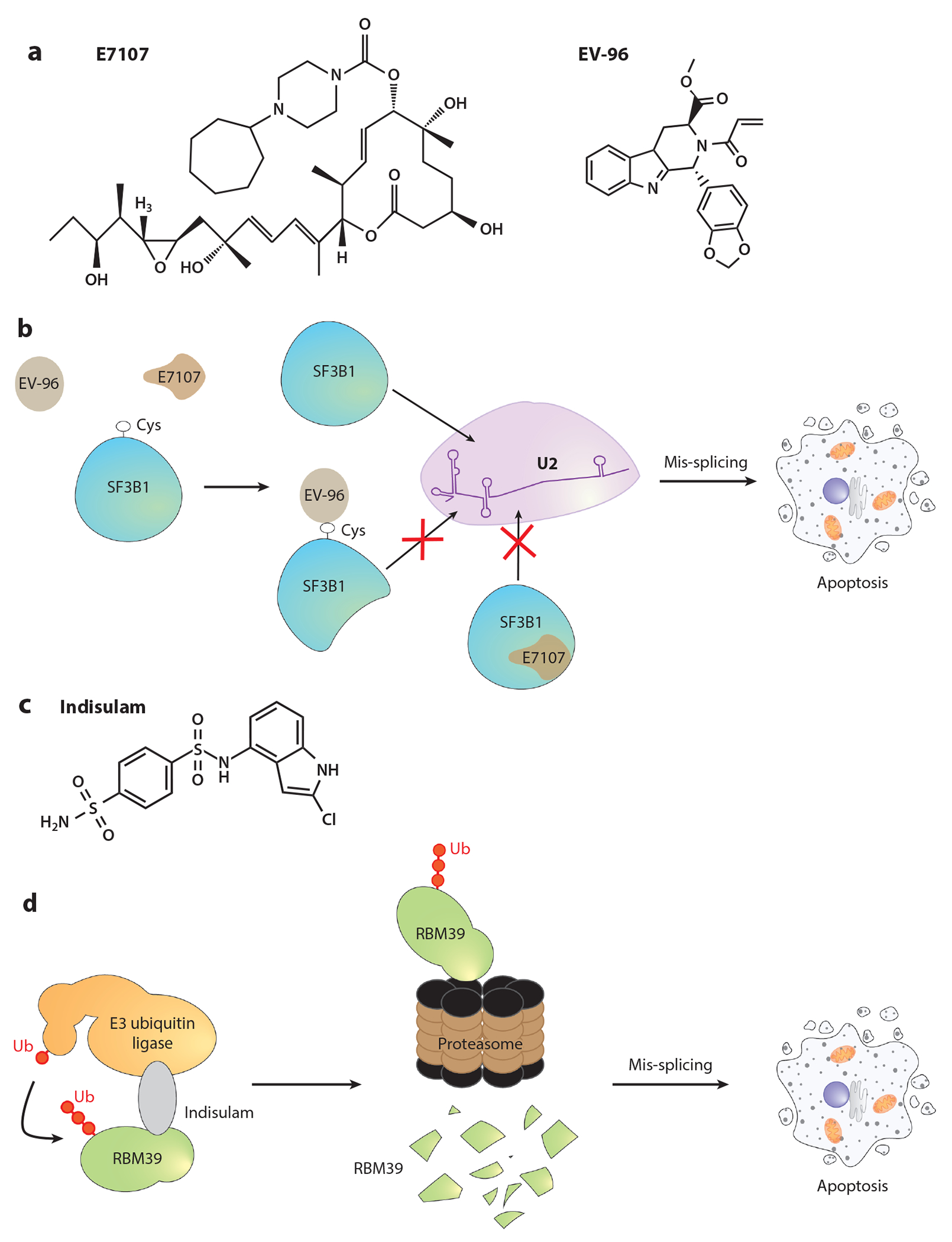
Mechanism of splicing factor 3b subunit 1 (SF3B1)-binding agents and RNA-binding motif protein 39 (RBM39) degraders. (*a*) Molecular structures of two SF3B1-binding drugs. (*Left*) E7107 is an SF3B1 noncovalent binding pladienolide. (*Right*) EV-96 is a tryptoline acrylamide that covalently binds a cysteine in SF3B1 (71). (*b*) SF3B1-binding agents prevent the U2 small nuclear ribonucleoprotein complex from binding precursor messenger and consequently result in global impairments in RNA splicing. (*c*) Structure of the RBM39 degrader indisulam, which is an aryl sulfonamide. (*d*) Aryl sulfonamides take advantage of the ubiquitin-proteasome system by binding RBM39 in the hydrophobic pocket and bringing the Cullin-RING E3 ubiquitin ligase 4 (CRL4) into physical proximity to RBM39, leading to its ubiquitination and degradation by the proteasome (75, 81). This leads to dysregulation of RNA splicing and drives apoptosis in cancer cells.

Pladienolide B was initially obtained naturally from *Streptomyces platensis* Mer-11107 and eventually led to the development of its synthetic urethane derivative and chemically stable analog E7107 (56–58) (Figure 1a). Pladienolides noncovalently bind SF3B1 to alter its conformation and disrupt splicing (59). Crystal and cryo-electron microscopy structures of Pladienolide B and E7107, respectively, complexed with SF3B showed that inhibition occurs specifically through preventing the complex from recognizing intronic branch point nucleotides (60, 61).

E7107 was evaluated in patients with cancer in two Phase I clinical trials with a total of 56 patients with different solid tumors refractory to available treatments. Unfortunately, clinical responses were not observed, and three patients developed ocular toxicity, leading to the cessation of further clinical investigation with E7107 (62, 63). Whether this ocular toxicity is attributed to the E7107 molecule itself or a general effect of potent SF3B1 inhibition is not well-understood (64, 65). It is noteworthy that E7107, GEX1A (also known as herboxidiene), and splicostatin A (SSA) all lead to a truncated version of the cell cycle inhibitor p27, which possesses resistance to typical proteolytic degradation due to its aberrantly truncated C-terminal domain (49, 58, 66).

H3B-8800 is an orally bioavailable semisynthetic analog of E7107 (67). Both E7107 and H3B-8800 have shown preclinical efficacy in myeloid leukemia models expressing mutant SF3B1 or SRSF2 (64, 68, 69). In a Phase I clinical trial with 84 patients with MDS, AML, or CMML, H3B-8800 was generally well-tolerated among patients with treatment-related adverse events, including nausea, vomiting, fatigue, and diarrhea. Unfortunately, this agent lacked efficacy and did not meet International Working Group criteria for partial or complete treatment response (70). While there is some evidence for SF3B target engagement in patients treated with H3B-8800, whether this inhibition was sufficient in potency and/or duration to result in clinical responses is unclear.

### Covalent SF3B1 Inhibitors

Beyond the natural product–derived SF3B noncovalent inhibitors described above, covalent inhibitors of SF3B1 have very recently emerged as a novel means to modulate RNA splicing. These agents offer prolonged inhibition by irreversibly modifying cysteine residues in SF3B1, locking the protein in an inactive state, and preventing its incorporation into the U2 snRNP and thus participation in spliceosomal assembly (71, 72) (Figure 1b). This strategy is potentially advantageous in cancers with high dependency for wild-type splicing activity, enabling sustained disruption of splicing in tumor cells while minimizing effects on normal tissues. The development of covalent inhibitors such as tryptoline acrylamides EV-96 and WX-02-23, while still in preclinical stages, represents a significant advancement and growing interest in targeting SF3B1 and its mutant variants in cancer, providing tools to address the limitations of earlier noncovalent inhibitors (Figure 1a). Similar to noncovalent inhibitors, EV-96 and WX-02-23 result in exon skipping and intron retention. Combination therapies involving covalent inhibitors and agents targeting complementary pathways, such as DNA damage repair inhibitors (such as PARP inhibitors) or apoptosis inducers (including venetoclax), are also under exploration. Early studies suggest that these combinations could enhance therapeutic efficacy and mitigate resistance mechanisms (71, 73).

EV-96 and WX-02-23 provide several advantages over their noncovalent counterparts, including the potential for enhanced potency and sustained inhibition of SF3B1 activity, while also targeting SF3B1 differently, thus offering an additional mechanism of treatment with similar results. Their irreversible binding reduces dosing frequency and may overcome adaptive resistance mechanisms as well as avoid toxicities associated with transiently binding noncovalent inhibitors (49, 71, 72). However, the irreversible nature of covalent inhibitors also introduces challenges. Off-target binding could result in unforeseen toxicity, especially in normal tissues with high spliceosome activity (74).

### RBM39 DEGRADERS

RNA-binding motif protein 39 (RBM39) is a critical regulator of RNA splicing and gene expression and plays an essential role in hematopoietic cells, maintaining normal cellular function (75, 76). RBM39 functions as a splicing cofactor, interacting with SF3B1 and U2AF2 to regulate premRNA splicing (75, 77, 78). RBM39 degraders, including aryl sulfonamide compounds such as indisulam, tasisulam, E7820, and chloroquinoxaline sulfonamide, exploit a unique mechanism to selectively degrade RBM39 through the ubiquitin-proteasome system (Figure 1c,d). This process is mediated by the Cullin-RING E3 ubiquitin ligase 4 (CRL4) E3 ubiquitin ligase complex, with the specific substrate receptor being the subunit, DDB1 and CUL4-associated factor 15 (DCAF15) (76, 78–80). These small molecules function as molecular glue degraders, inducing a physical interaction between RBM39 and DCAF15. Aryl sulfonamide molecules are thought to bind to a hydrophobic pocket on RBM39 while the opposite end of the compound binds to DCAF15, forming a ternary complex that brings the two proteins within close proximity (75, 81). The CRL4-DCAF15 complex catalyzes the addition of ubiquitin moieties to specific residues on RBM39. Subsequently, ubiquitinated RBM39 is recognized and degraded by the proteasome, leading to dysregulation of RNA splicing and apoptosis in cancer cells dependent on this pathway (77, 82).

RBM39 degraders have shown promise in a number of cancer preclinical studies. However, in Phase II clinical trials of indisulam in unselected patients with solid tumors refractory to standard therapy, monotherapy was not enough to elicit a response (77, 83). In breast cancer, indisulam and tasisulam demonstrated strong preclinical activity, including growth inhibition and cell cycle arrest in cancer cell lines and patient-derived xenograft (PDX) models, but in two Phase II clinical trials, indisulam showed limited therapeutic benefits (NCT00165880), and tasisulam demonstrated stabilization or regression of disease in less than 10% of patients (NCT00992225) (77, 80). For melanoma, tasisulam showed initial promise in early-phase trials but faced significant safety concerns, including adverse events in Phase III trials, which led to its discontinuation despite evidence of antiproliferative effects in preclinical studies (77). Aryl sulfonamides have progressed through nearly 50 Phase I and II trials to date, with varying efficacy in solid tumors (82). The majority of these trials demonstrated manageable toxicity profiles and minor to moderate antitumor activity in select patient populations.

Given knowledge that cancers with neomorphic change of function mutations in the RNA splicing machinery are preferentially dependent on otherwise wild-type splicing catalysis and preclinical studies demonstrating sensitivity of leukemia cells with these mutations to RBM39 degradation (84), Bewersdorf et al. (83) carried out the first trial of an RBM39 degrader specifically in myeloid leukemia patients with a splicing factor mutation. This study was a Phase II clinical trial of the oral RBM39 degrader E7820 as a single agent specifically in patients with MDS and AML that had relapsed or was refractory to approved therapies and with a mutation in SF3B1, SRSF2, U2AF1, or ZRSR2. The E7820 dose used was established based on the maximum tolerated dose in prior Phase I studies in solid tumor patients. This dose was well-tolerated in patients, and for the first time, RBM39 degradation and drug-induced splicing changes were documented in patients treated with an RBM39 degrader. At the same time, there were no clinical responses as a single agent, and the degree of target engagement (as assessed by Western blotting of RBM39 and RNA sequencing in peripheral blood mononuclear cells from patients in the study) was modest compared to that achieved with E7820 in preclinical models where efficacy was evident. As such, this study identified that RBM39 degraders may not have achieved adequate clinical responses in patients due to incomplete RBM39 degradation in vivo.

Recent studies have shown that patients with homologous recombination deficiency and/or cancers possessing high levels of v-myc avian myelocytomatosis viral oncogene neuroblastoma derived homolog (MYCN) may strongly benefit from RBM39 degraders (76, 80). However, further characterization of biomarkers indicative of splicing profiles or RBM39 dependency is needed to identify patients who would benefit most from these therapies.

## PRMT5 INHIBITORS

### *S*-Adenosylmethionine-Competitive PRMT5 Inhibitors

Protein arginine methyltransferases (PRMTs) are critical enzymes that regulate various cellular processes through the methylation of arginine residues. Methyl arginine modifications influence chromatin remodeling, transcription, RNA splicing, and DNA damage repair, among other functions. PRMTs are categorized into three types based on their methylation products: Type I PRMTs catalyze asymmetric dimethylation of arginines, type II PRMTs catalyze symmetric dimethylation, and type III PRMTs catalyze monomethylation only (85). PRMT5, a type II enzyme, is particularly significant in cancer biology. PRMT5 symmetrically dimethylates arginine residues on spliceosomal components, facilitating snRNP assembly. This activity is crucial for maintaining RNA splicing and cellular homeostasis (86–88).

PRMT5 overexpression is strongly linked to several cancers, including but not limited to certain subtypes of AML, lung cancer, colorectal cancer, glioblastoma, and breast cancer (87). Cancer cells exhibit heightened dependency on PRMT5-mediated processes such as RNA splicing and transcription repression, making these pathways vulnerable to therapeutic intervention. PRMT5 inhibition not only disrupts splicing of oncogenic transcripts but also reactivates tumor suppressor genes by reversing histone H4R3 methylation. For instance, PRMT5-mediated methylation suppresses tumor suppressors, and PRMT5 inhibitors effectively relieve this repression, restoring antitumorigenic gene expression (87, 89).

**Figure 2:**
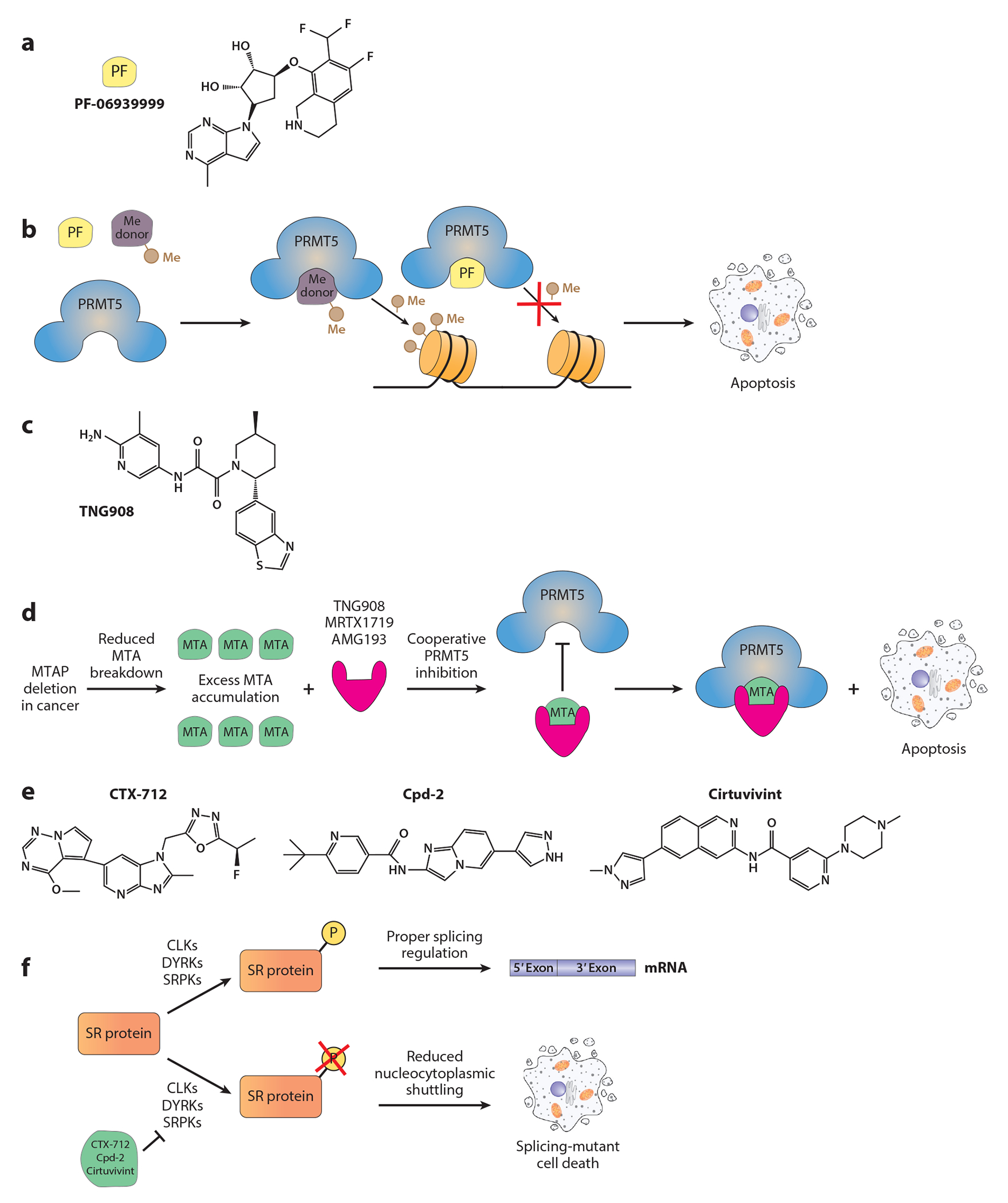
Mechanisms of action for inhibitors of enzymes that catalyze posttranslational modifications on RNA splicing factors. (*a*) Structure of PF-06939999, an *S*-adenosylmethionine (SAM)-competitive protein arginine methyltransferase 5 (PRMT5) inhibitor. (*b*) SAM-competitive PRMT5 inhibitors such as PF-06939999 mimic SAM and bind to the methyl donor (Me donor) site of PRMT5, leading to a reduction in the methylation of targets such as arginine residues on histones and RNA splicing factors. (*c*) Structure of TNG908, a brain-penetrant methylthioadenosine (MTA)-cooperative PRMT5 inhibitor. (*d*) Methylthioadenosine phosphorylase (MTAP) deletion leads to impaired metabolism of MTA, resulting in an excess accumulation of intracellular MTA. This excess MTA can synergize with an MTA-cooperative PRMT5 inhibitor to compete with SAM for PRMT5 binding, leading to a reduction in methylation capabilities for substrates such as splicing factors and histones and resulting in MTAP-null cell death. (*e*) Structures of the CMGC kinase family Cdc-like kinase (CLK) inhibitor CTX-712, the CLK/serine-rich protein kinase (SRPK) inhibitor Cpd-2, and the CLK/dual-specific tyrosine-regulated kinase (DYRK) inhibitor cirtuvivint. (*f*) CLKs, DYRKs, and/or SRPKs phosphorylate serine-arginine rich (SR) proteins such as SRSF1, leading to nucleocytoplasmic shuttling and regulation of splicing. Inhibiting SRSF1 phosphorylation impairs nucleocytoplasmic shuttling of SRSF1 and results in global changes in RNA splicing.

*S*-adenosylmethionine (SAM)-competitive inhibitors target PRMTs by mimicking SAM and binding to the methyl donor site, thereby blocking the methylation of target proteins. This inhibition disrupts downstream processes such as RNA splicing and transcriptional regulation, leading to cancer cell death. PRMT5 inhibitors such as PF-06939999 work by inhibiting SAM binding, thus hindering the enzyme’s ability to methylate substrates (Figure 2a,b). However, more recently, PRMT5 inhibitors like JNJ-64619178 not only bind the SAM-binding site but also extend into the substrate-binding pocket, enhancing specificity and potency (88, 90).

PF-06939999 is a highly selective PRMT5 inhibitor that effectively reduces symmetric dimethylarginine levels in cancer cells. By targeting PRMT5’s catalytic activity, it disrupts spliceo-some functionality, inducing splicing errors in key oncogenic transcripts such as MYC, CDK4, and BCL-2L1. This leads to NMD and apoptosis of cancer cells, particularly in NSCLC models (85, 87). Similarly, JNJ-64619178 acts as a dual SAM-and substrate-competitive inhibitor, showing promising preclinical activity in both solid tumors and hematologic malignancies (85, 89).

SAM-competitive PRMT5 inhibitors have shown mixed results as a monotherapy to date. JNJ-64619178 underwent Phase I trials for patients with diffuse large B cell lymphoma, low-risk MDS, and malignant solid tumors (NCT03573310), demonstrating manageable toxicity but only achieving a response rate of approximately 6% (85, 89). Additionally, in a Phase I trial for JNJ-64619178 in 90 patients to treat advanced malignant solid tumors and non-Hodgkin lymphomas, thrombocytopenia was the dose-limiting toxicity, and the drug only achieved an objective response rate of 5.6% (91). While preclinical trials of PF-06939999 highlighted the drug’s ability to selectively induce splicing errors in cancer cells with high PRMT5 dependency, including those harboring splicing factor mutations, the Phase I clinical trial (NCT03854227) saw no clinical response (85, 87, 92). Another Phase I trial for PF-06939999 for patients with advanced or metastatic solid tumors demonstrated tolerable safety and achieved partial responses in 3 patients with either head and neck squamous cell carcinoma or NSCLC and stable disease in 19 patients (92).

To date, the only clinical trial to have evaluated PRMT5 inhibition in patients with an RNA splicing factor mutation [a genetic subtype of cancer shown to be preferentially sensitive to inhibition of PRMT5 or type I PRMTs (93)] has demonstrated some evidence of clinical benefit. This was a Phase I dose-escalation and dose-expansion trial of the SAM-competitive PRMT5 inhibitor PRT-543 evaluating the safety and efficacy of monotherapy of this drug in patients with relapsed/refractory MDS, AML, and MDS/myeloproliferative neoplasms overlap with a mutation in SF3B1, SRSF2, U2AF1, or ZRSR2 (94). This study enrolled 40 patients across the spectrum of RNA splicing mutations, and a number of patients achieved clinical responses. This included marrow complete remission in one patient (3.0%) and hematologic improvement in three patients (9.1%), and one AML patient (14.3%) achieved a complete remission with incomplete hematologic recovery with clearance of all baseline somatic mutations. Interestingly, nearly all responding patients had an SRSF2 mutation specifically. Moreover, an additional Phase I trial of the SAM-uncompetitive PRMT5 inhibitor GSK3326595 as a monotherapy for 30 patients with relapsed or refractory myeloid neoplasms (AML, MDS, CMML) found that of the 30 patients treated, three of the five patients with clinical benefit had an SRSF2 mutation (95). These studies suggest a specific potential link between mutations in SRSF2 and response to PRMT5 inhibition. Determining the mechanistic basis for this association and performing focused future Phase II studies specifically in SRSF2 mutant patients (and potentially in combination with other agents) may result in promising clinical responses in this high-risk group of patients with myeloid malignancies.

### Methylthioadenosine-Cooperative PRMT5 Inhibitors

First-generation SAM-competitive PRMT5 inhibitors showed indiscriminate PRMT5 inhibition between normal and malignant cells, leading to on-target toxicity characterized by cytopenias in patients in clinical trials conducted thus far (96). Methylthioadenosine (MTA) is an analog of SAM, and MTA-cooperative PRMT5 inhibitors appear to have great potential in treating methylthioadenosine phosphorylase (MTAP)-deleted cancers, which have approximately a 10% prevalence in all cancers. Homozygous MTAP deletion is recurrent across leukemias and lymphomas, as well as lung, brain, bone, breast, skin, mesothelial, squamous cell, pancreatic, biliary tract, prostate, and bladder cancers (97). Notably, MTAP deletion leads to the accumulation of intracellular MTA. When treated with an MTA-cooperative PRMT5 inhibitor, MTA competes with SAM to produce the PRMT5-methylosome protein 50 (MEP50)/MTA complex in MTAP-deleted tumors, which could be targeted by a PRMT5-MEP50/MTA complex inhibitor (98–100) (Figure 2*c*,d).

TNG908 is a brain-penetrant MTA-cooperative PRMT5 inhibitor that exhibits lethality to MTAP-deleted cancers in mouse xenograft models (101). TNG908 selectively kills MTAP-deleted cells at a 15-times-higher rate than wild-type cells with MTAP intact. TNG908 is being tested in an ongoing Phase I/II clinical trial in patients with advanced or metastatic glioblastoma, sarcoma, or mesothelioma (NCT05275478) (101, 102).

MRTX1719 is another MTA-cooperative PRMT5 inhibitor with demonstrated efficacy in several xenograft models of MTAP-deleted cancers (103, 104). An ongoing Phase I/II clinical trial for MRTX1719 to treat relapsed or refractory MTAP-deleted solid tumors has shown complete PRMT5-dependent inhibition of symmetric dimethylation of arginine in neoplastic cells from three patients, objective tumor responses in six patients, and no dose-limiting adverse events in any of the patients (NCT05245500) (104).

AMG 193 has demonstrated in vitro efficacy for MTAP-deleted cancers by inducing cell cycle arrest in the G2/M phase, DNA damage, and aberrant RNA splicing (105, 106). AMG 193 additionally seems to have synergistic effects when used in combination with chemotherapies or the Kirsten rat sarcoma virus (KRAS) G12C inhibitor sotorasib in vivo, and additionally, sensitivity to treatment in vitro correlates with MTAP loss. AMG 193 has demonstrated promise in the early stage of a Phase I clinical trial with 80 patients through safety profiles and an objective response rate of 21% across eight different cancers (105). Dose-limiting toxicities were present in eight patients and included nausea, vomiting, fatigue, hypersensitivity reaction, and hypokalemia. AMG 193 is additionally being investigated regarding its efficacy in MTAP-deleted solid tumors in combination with chemotherapy or immunotherapy (NCT06333951 and NCT06360354) (106).

## KINASE INHIBITORS WITH IMPACTS ON RNA SPLICING

Proper assembly and subcellular localization of splicing factors is regulated by phosphorylation as well as arginine methylation of a number of splicing proteins (Figure 2e,f). A number of kinases are known to prominently phosphorylate splicing factors, including members of the CMGC kinase family Cdc-like kinases (CLKs), dual-specific tyrosine-regulated kinases (DYRKs), and serine-rich protein kinases (SRPKs) (107–109). CLK and DYRK enzymes are dual-specificity (tyrosine and serine/threonine) kinases, which phosphorylate proteins involved in premRNA splicing, and genetic or pharmacologic inhibition of these kinases disrupts splicing globally (110). SRPKs are serine/threonine kinases that phosphorylate SR proteins. Nuclear CLK and SRPK proteins work together to regulate SR protein subcellular localization through phosphorylation between nuclear speckles and the nucleoplasm (111, 112). Dysregulation of CLK, DYRK, and SRPK activity can lead to aberrant splicing, and thus their inhibition is a promising therapeutic target (113).

### CLK and DYRK Inhibitors

ATP-competitive inhibitors of CLKs have been developed and modulate CLK-dependent SRSF2 splicing activity while also suppressing SR protein phosphorylation, resulting in nuclear speckle dissociation and cell death (114, 115) (Figure 2e). The benzothiazole compound TG003 demonstrated potent inhibition of CLK1, CLK2, and CLK4, laying the foundation for future CLK inhibitor therapeutic development (107). T-025 treatment has shown promising antitumor effects in MYC-driven breast cancer models in vivo (116). Furthermore, preclinical studies have demonstrated the potency of Cpd-1, Cpd-2, and Cpd-3 as inhibitors of both CLKs and SRPKs (117). The CLK inhibitors 1C8 and GPS167 showed antitumor efficacy in vitro in a wide range of cell lines through their impact on AS in several areas, including mitotic spindle assembly components, antiviral immune response, MYC targets, and the epithelial-mesenchymal transition (118).

A Phase I clinical trial in Japan of the CLK inhibitor CTX-712 in 14 patients with re-lapsed/refractory AML and high-risk MDS has demonstrated an acceptable safety profile with an overall response rate of 42.9%, including four patients with complete remissions (119, 120). This is an impressive response rate, and it will be important to see whether similar activity is observed in the ongoing Phase I trials of this agent in the same patient population in the United States (NCT05732103). Importantly, the responses seen with CLK inhibition in preclinical studies of myeloid leukemias do not appear to be limited to those with RNA splicing factor mutations (121, 122). Several clinical trials of additional CLK inhibitors are ongoing and include studies with SM08502 (cirtuvivint) and BH30236 for solid tumors or hematologic malignancies (123– 126). Cirtuvivint is a CLK inhibitor that has shown safety and efficacy in a Phase I clinical trial for prostate cancer and in preclinical studies in AML has shown promise in synergizing with venetoclax and overcoming venetoclax resistance (122, 127). A Phase I study combining cirtuvivint with the oral DNA hypomethylating agent ASTX727 in patients with relapsed/refractory MDS and AML has recently begun (NCT06484062).

The existing CLK inhibitors noted above all inhibit multiple CLKs (with most inhibiting CLK1–4) as well as the related family of DYRKs (DYRK1A/B and DYRK2–4). For example, cirtuvivint is an ATP-competitive inhibitor of CLK1–4 and DYRK1–4, while BH30236 inhibits DYRK1/2, PIM3, and FLT3 in addition to CLK3. Currently it is unclear whether inhibiting individual CLK or DYRK protein members is important for the therapeutic activity and/or toxicity of these agents.

### SRPK Inhibitors

SRPK1 is a serine/threonine kinase that plays an important role in RNA splicing by regulating the intranuclear distribution of splicing factors and nuclear speckle reorganization during interphase and mitosis, respectively (128). Several pro-oncogenic RNA isoforms encoding cancer-promoting proteins have been described as being regulated by SRPK1 activity, including SRPK controlling a splicing switch from pro- to antiangiogenic vascular endothelial growth factor (VEGF) isoforms. As such, SRPK1 inhibition has previously been demonstrated to impair tumor angiogenesis (129– 132). A newly developed SRPK inhibitor, C-DBS, covalently binds to the SRPK-specific docking groove and leads to the impairment of SR protein phosphorylation, angiogenesis, cell migration, and cell invasion (133). It will be exciting to identify whether SRPK1 inhibition can have a clinical impact in the near future, as to date no SRPK inhibitors have been evaluated in a clinical setting.

## UHM-BINDING DRUGS

**Figure 3:**
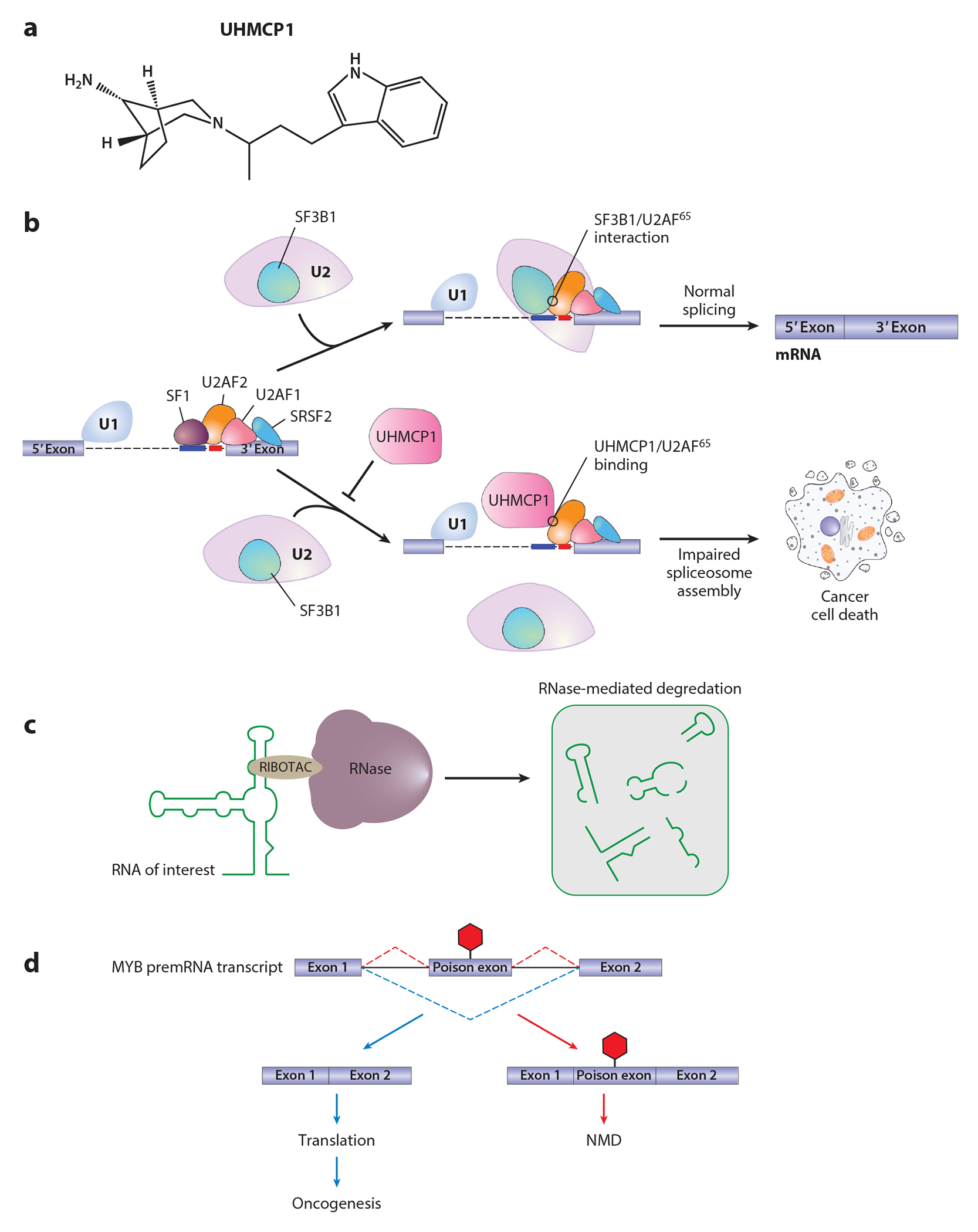
Mechanisms of action for U2AF homology motif (UHM)-binding drugs, RNA-targeting chimeras (RIBOTACs), and myeloblastosis (MYB) degradation. (*a*) Structure of the UHM domain–binding agent UHMCP1. (*b*) During normal spliceosome assembly, the U2 small nuclear RNA auxiliary factor (U2AF) ligand motif in the N terminus of splicing factor 3b subunit 1 (SF3B1) interacts with U2AF’s 65-kDa subunit, U2AF^65^, in the UHM-binding domain. This leads to normal splicing and mature messenger RNA (mRNA). UHMCP1 disrupts the SF3B1/U2AF^65^ interaction by binding the UHM domain of U2AF^65^ in its hydrophobic pocket to prevent spliceosome assembly (135). This slows down splicing kinetics, influences alternative splicing, and reduces cellular viability. (*c*) The mechanism for RIBOTACs such as Cugamycin. The RIBOTAC acts as a bridge between an RNase and an RNA of interest, leading to RNase-mediated decay of the transcript (138, 157). (*d*) The MYB mRNA degrader REM-422 works by binding to MYB precursor messenger RNA (premRNA) and inducing aberrant inclusion of a poison exon that would normally be excised with the intron. MYB poison exon inclusion results in reduced MYB mRNA and protein via subsequent nonsense-mediated decay (NMD) of the MYB transcript.

As described above, U2AF1 is a core component of the splicing machinery that heterodimerizes with U2AF2 through its U2AF homology motif (UHM) domain to recognize the 3′AG dinucleotide in the 3′ splice site. The C-terminus of the UHM domain in U2AF2, in turn, interacts with the N-terminal U2AF ligand motif of SF1, leading to SF3B complex recruitment to the intronic branch site (10). The identification of small-molecule phenothiazine derivatives that bind to the hydrophobic pocket of the UHM domain to impair protein-protein interactions has demonstrated another therapeutic avenue for the inhibition of splicing (134, 135). UHMCP1 is one such molecule that disrupts SF3B155/U2AF65 interactions and influences AS to lead to reduced cellular viability (135) (Figure 3a,b). A recent comprehensive analysis of UHM domain inhibition highlights the development of SF1–8, which showed in vitro efficacy in killing leukemic cell lines expressing mutant U2AF1 and primary human MDS cells with a U2AF1 mutation (136). Conversely, a stabilizing assembly small molecule, NSC194308, works to preferentially kill U2AF1 mutant cancer cells by enhancing spliceosome assembly at an early-stage U2AF2-RNA splice site complex and then stalling further splicing through its hydrophobic and electrostatic moieties that keep the complex bridged together (137). These studies highlight the therapeutic potential of enhancing and stabilizing the spliceosome to selectively target splicing factor mutant cancer cells.

## RNA-TARGETING THERAPEUTICS

Most small molecules are typically designed to target proteins of interest, but due to a lack of currently druggable protein moieties specific to cancer (such as active sites, cysteine residues, and allosteric binding sites), there has been interest in targeting RNA directly. By focusing therapeutics on targeting the unique and highly defined sequences of nucleotides of RNA, such an approach may alleviate limitations in protein specificity and the need to understand highly complex protein interactions in vivo. To date there have been a number of drug classes used to target RNA, namely antisense oligonucleotides (ASOs), small-molecule inhibitors, and RNA-targeting chimeras (RIBOTACs). These approaches hold promise for numerous diseases driven by aberrant splicing, transcriptional dysregulation, or the overexpression of pathogenic transcripts, including cancers (138–141).

### Oligonucleotides

ASOs are short, synthetic single-stranded nucleic acids designed to hybridize with target RNAs through Watson-Crick base-pairing. These molecules exert their effects by inducing RNA degradation via RNase H activation, steric blocking of RNA translation, or modulation of splicing patterns. Splice-switching oligonucleotides (SSOs), a subset of ASOs, hybridize in a sequence-specific manner to create steric hindrance at critical *cis* regulatory sequences, secondary RNA structures, or splice sites. By preventing the recruitment of splicing factors without cleaving the RNA, SSOs leave total mRNA levels unaffected, unlike small interfering RNAs. However, ASOs face challenges such as an inability to cross the blood-brain barrier and limited stability (142–145).

SSOs have been useful pharmacologic tools to probe the impact of splice inhibition and activation in cancer preclinical studies. In splice inhibition, SSOs block cryptic splice sites to restore proper protein isoforms, correct reading frames, or delete toxic sequences via exon skipping (142–145). For example, BRD9 mis-splicing in cancers driven by SF3B1 mutations can be addressed by blocking poison exon inclusion, restoring BRD9 protein expression and reducing tumor growth (146). Similarly, signal transducer and activator of transcription 3 (STAT3) exon 23 skipping promotes a shift from the pro-oncogenic α isoform to the tumor-suppressive β isoform, increasing cell death and reducing tumor progression. Another approach, BCL-X exon 6 skipping, promotes the proapoptotic BCL-XS isoform, which has been shown to reduce tumor size in preclinical models (142, 145).

Splice activation by SSOs can upregulate splicing enhancer proteins or block splicing inhibitors to promote exon inclusion. In spinal muscular atrophy (SMA), early efforts targeting the 3′ splice site of exon 8 evolved to targeting *cis* regulatory elements of exon 7, such as activating the intronic splicing silencer N1, which interacts with the exon 7 repressive proteins hnRNPA1/A2. These concepts led to the development of nusinersen, the first US Food and Drug Administration (FDA)-approved SSO for SMA, which enhances exon 7 inclusion in SMN2 premRNA to increase functional survival motor neuron (SMN) protein levels and prolongs the survival of infants with type 1 SMA (147, 148).

FDA-approved ASOs such as eteplirsen, golodirsen, and casimersen target specific exons in the dystrophin gene to restore its reading frame for patients with Duchenne muscular dystrophy (147, 149). Although clinical trials for ASOs targeting oncogenic transcripts, such as those modifying BCL-2 splicing in combination with chemotherapy, have demonstrated their safety, efficacy has been limited (145, 150). Advances in chemical modifications, such as locked nucleic acids and phosphorothioate backbones, continue to improve ASO stability, specificity, and delivery, holding immense promise for expanding ASO applications in oncology.

### Small Molecules and Degraders

Beyond nucleic acid therapeutics, small molecules are being designed to bind RNA to selectively exploit its unique Watson-Crick sequence information and structural motifs (151, 152). As a result, such small molecules can be used in a wide range of applications, including prohibiting the production of undruggable proteins, impeding the function of specific structural regions of the RNA, and even helping to direct splicing. Two such directed splicing small molecules, risdiplam and branaplam, underwent clinical trials for patients with SMA (153). While branaplam was ultimately discontinued during Phase II clinical trials due to toxicity, risdiplam was the first RNA-targeting small molecule to be approved by the FDA. Risdiplam has been shown to act as a molecular glue, helping bridge the RNA to the U1 snRNP allowing for exon 7 retention and an increase in protein levels of SMN, essential for patients suffering from SMA. This molecule opened the door to many novel strategies and small molecules to target RNA in the context of cancer.

One of the newest RNA-targeting modalities is RIBOTACs (141, 151, 152, 154–156). Spearheaded by the Childs-Disney group (138, 157), RIBOTACs, such as risdiplam, act as a molecular glue between RNA and a protein of interest; however, unlike risdiplam, RIBOTACs, such as targaprimir-515 and Cugamycin, seek to couple RNA to ribonuclease recruiters, enabling selective degradation of disease-causing RNAs in breast cancer and myotonic dystrophy type 1, respectively (Figure 3c). Remix Therapeutics has also created a potent molecule targeting the transcript from c-Myb, a key oncogene in several hematologic malignancies, including AML (140, 158, 159), for degradation. This agent works by binding to MYB mRNA and inducing the aberrant inclusion of a poison exon, thereby promoting degradation of MYB mRNA via NMD (Figure 3d). REM-422 has demonstrated preclinical efficacy in reducing tumor cell viability in AML models and is under evaluation in Phase I clinical trials in AML (NCT06297941) and adenoid cystic carcinoma (NCT06118086) (160). Clinical investigations are ongoing to evaluate REM-422 in other MYB-dependent malignancies.

## FUTURE DIRECTIONS AND CONCLUSIONS

While many therapeutic agents modulating RNA splicing have been described and are currently in preclinical or early-phase clinical development, to date, no therapies that modulate splicing have received FDA approval for the treatment of any cancer. One major question in the use of agents that globally modulate splicing is whether there will be a safe therapeutic index given that RNA splicing is an essential process. To this end, several Phase I studies have demonstrated target engagement in vivo with evidence of modulation of RNA splicing in target tissues in patients and at acceptable toxicities (70, 83, 94). However, in each case, it is not clear that the extent of splicing modulation achieved in patients is sufficient to achieve therapeutic benefit and at a level commensurate with those seen in preclinical studies.

Several recent studies have highlighted that pharmacologic modulation of RNA splicing (161), and even endogenous alterations in RNA splicing within cancer cells (162, 163), generates novel antigens with immunogenic potential. As such, developing chemical means to potently and safely alter RNA splicing in patients with cancer could have enormous potential. Importantly, however, there has yet to be a clinical study evaluating the potential synergistic effect of combining splicing modulators with immunotherapeutic agents such as immune checkpoint inhibitors. Such a study would also be critical to finally address potential off-tumor autoimmune effects of such a combination in patients.

One major motivation for developing drugs to globally modulate RNA splicing in cancer was the discovery of cancers with neomorphic mutations in the RNA splicing machinery that are known to be highly sensitive to splicing perturbation. Although no splicing modulatory agents have demonstrated success in targeting these cancers in clinical studies to date, our group has recently developed synthetic RNAs that are uniquely regulated by cells with splicing factor mutations (164, 165). These synthetic introns are used to regulate the expression of therapeutic payloads that are selectively expressed and can selectively eliminate cancer cells. Developing means to effectively deliver these RNAs in vivo to tumor cells could have great therapeutic potential. Similarly, improved RNA delivery approaches could facilitate the use of other nucleic acid therapies in cancer.

## Supplementary Material

Supplementary Table 1

## Abbreviations

Alternative splicing (AS): a cellular process by which exons from the same gene are joined in different combinations
Exonic splicing enhancers (ESEs): nucleotides within exons that are bound by RNA-binding proteins that promote spliceosome assembly adjacent to that region on RNA
Exonic splicing silencers (ESSs): nucleotides within exons that are bound by RNA-binding proteins that repress spliceosome assembly adjacent to that region on RNA
Intronic splicing enhancers (ISEs): nucleotides within introns that are bound by RNA-binding proteins that promote spliceosome assembly adjacent to that region on RNA
Intronic splicing silencers (ISSs): nucleotides within introns that are bound by RNA-binding proteins that repress spliceosome assembly adjacent to that region on RNA
Serine-arginine rich (SR): a motif of amino acids with frequent serine-arginine dipeptides that is commonly found in RNA-binding proteins and splicing factors
Heterogeneous nuclear ribonucleoproteins (hnRNPs): a family of RNA-binding proteins that are most frequently associated with repression of alternative RNA splicing
Protein arginine methyltransferases (PRMTs): a family of enzymes that catalyze the posttranslational modification of placing an arginine on protein substrates
*S*-adenosylmethionine (SAM): a common cosubstrate utilized by enzymes to transfer methyl groups as well as catalyze transsulfuration and aminopropylation
Methylthioadenosine (MTA): an *S*-methyl derivative of the adenosine that serves as an intermediate in the methylthioadenosine cycle
Methylthioadenosine phosphorylase (MTAP): an enzyme that catalyzes the first step in the salvage of both adenine and methionine
CMGC kinase family Cdc-like kinases (CLKs): a family of dual-specificity kinases, related to DYRK kinases, which catalyze phosphorylation of both tyrosine and serine/threonine residues
Dual-specific tyrosine-regulated kinases (DYRKs): a family of dual-specificity kinases, related to CLK kinases, which catalyze phosphorylation of both tyrosine and serine/threonine residues
Serine-rich protein kinases (SRPKs): a family of kinases that phosphorylate serine residues and are best characterized as phosphorylating SR proteins
U2AF homology motif (UHM): an amino acid motif that is important in mediating protein-protein interactions with U2AF ligand motifs
Antisense oligonucleotides (ASOs): single- or double-stranded oligonucleotides that are chemically modified to increase stability and bind substrate RNA to regulate splicing or decay
RNA-targeting chimeras (RIBOTACs): molecules that bind an RNA target and degrade it by recruiting endogenous RNases
Splice-switching oligonucleotides (SSOs): modified nucleic acids that base-pair with a premRNA and disrupt its normal splicing
Spinal muscular atrophy (SMA): neuromuscular disorder that results in the loss of motor neurons and progressive muscle wasting due to inherited mutation in *SMN1*
Survival motor neuron (SMN): protein encoded by *SMN1* and *SMN2* genes and involved in the assembly of the RNA-protein complexes of the spliceosome
