# Supplementary material for: RNA Splicing as a Therapeutic Target in Cancer
Source: Annu Rev Pharmacol Toxicol. Author manuscript; Available in PMC 2026 Jun 21. (PMC13283465; doi:10.1146/annurev-pharmtox-062124-035809)
Supplement: Supplementary Table 1 [file NIHMS2180747-supplement-Supplementary_Table_1.pdf]

Downloaded from www.annualreviews.org. Guest (guest) IP: 74.254.78.132 On: Mon, 08 Jun 2026 14:08:54

Supplemental Table 1. Stage of development for cancer therapeutics impacting RNA splicing.

Target	Class	Agent	Clinical Phase	Patient Population	Reference
Noncovalent SF3B1 Binding	Spliceostatins	FR901463, FR901464, and FR901465	Preclinical	NA	(1; 2)
		Spliceostatin A	Preclinical	NA	(3)
		Meayamycin B	Preclinical	NA	(4)
		Sudemycins C-F	Preclinical	NA	(5; 6)
	Herboxidienes	GEX1A	Preclinical	NA	(7)
		6-Norherboxidiene	Preclinical	NA	(8)
	Pladienolides	Pladienolides A-G	Preclinical	NA	(9; 10)
		E7107	1	Advanced or metastatic solid tumors	(11; 12)
		H3B-8800	1	MDS, CMML, AML	(13; 14)
Covalent SF3B1 Binding	Tryptoline Acrylamides	EV-96	Preclinical	NA	(15)
		WX-02-23	Preclinical	NA	(16)
RBM39 Degradation	Aryl Sulfonamides	Indisulam	2	R/R AML, HR-MDS	(17)
		Tasisulam	2	Unresectable or metastatic melanoma	(18)
		E7820	2	R/R splicing factor mutant AML, MDS, or CMML	(19)
		Chloroquinoxaline Sulfonamide	2	NSCLC	(20)

Downloaded from www.annualreviews.org. Guest (guest) IP: 74.254.78.132 On: Mon, 08 Jun 2026 14:08:54

PRMT5 Inhibition	SAM Inhibitors	PF-06939999	1	Advanced or metastatic solid tumors	(21; 22)
		JNJ-64619178	1	R/R B-cell NHL, LR-MDS	(23)
		PRT-543	1	R/R splicing factor mutant MDS, AML, MDS/MPN	(24)
		GSK3326595	1	R/R MDS, CMML, AML	(25)
	MTA-Cooperative Inhibitors	TNG908	1/2	Advanced or metastatic MTAP-deleted solid tumors (including glioblastoma)	(26; 27)
		MRTX1719	1/2	Advanced, unresectable, or metastatic MTAP-deleted solid tumors	(28)
		AMG 193	1	Advanced or metastatic MTAP-deleted solid tumors	(29)
Kinase Inhibition	CLK and DYRK Inhibitors	TG003	Preclinical	NA	(30)
		T-025	Preclinical	NA	(31)
		1C8	Preclinical	NA	(32)
		GPS167	Preclinical	NA	(32)

Downloaded from www.annualreviews.org. Guest (guest) IP: 74.254.78.132 On: Mon, 08 Jun 2026 14:08:54

		CTX-712	1/2	R/R AML, HR-MDS, MDS/MPN	(33; 34)
		BH30236	1	R/R AML, HR-MDS	(35), NCT06501196
		Emavusertib (CA-4948)	1/2	LR-MDS with anemia, MRD+ AML patients in CR or CRh, R/R AML, HR-MDS	(36), NCT05178342
		SM08502 (Cirtuvivint)	1	Advanced solid tumors, R/R AML and MDS,	(37; 38)
	SRPK Inhibition	C-DBS	Preclinical	NA	(39)
		SPHINX31	Preclinical	NA	(40)
		SRPIN340	Preclinical	NA	(40)
	CLK and SRPK Inhibition	Cpd-1, Cpd-2, Cpd-3	Preclinical	NA	(41)
UHM Binding	Phenothiazine Derivatives	UHMCP1	Preclinical	NA	(42)
		NSC194308	Preclinical	NA	(43)
RNA Degradation	RIBOTAC	Targaprimir-515	Preclinical	NA	(44)
		Cugamycin	Preclinical	NA	(45)
	MYB Degradation	REM-422	1	R/R AML, HR-MDS, advanced or metastatic ACC	(46) NCT06297941, NCT06118086

Downloaded from www.annualreviews.org. Guest (guest) IP: 74.254.78.132 On: Mon, 08 Jun 2026 14:08:54

Abbreviations: ACC, adenoid cystic carcinoma; AML, acute myeloid leukemia; CMML, chronic myelomonocytic leukemia; CLK, Cdc-like kinase; CR, complete remission; CRh, complete remission with partial hematologic recovery; DYRK, dual-specificity tyrosine-regulated kinase; HR-, high-risk; LR-, low-risk; MDS, myelodysplastic syndromes; MDS/MPN, myelodysplastic/myeloproliferative neoplasms; MRD+, minimal residual disease positive; MTAP, methylthioadenosine phosphorylase ; MTA, methylthioadenosine; NA, not applicable; NHL, non-Hodgkin's lymphoma; NSCLC, non-small cell lung cancer; PRMT5, protein arginine methyltransferase 5; RBM39, RNA-binding motif protein 39; RIBOTAC, ribonuclease targeting chimeras; SAM, S-adenosylmethionine; SRPK, serine/arginine-rich protein kinase; SF3B1, splicing factor 3B subunit 1; UHM, U2AF homology motif; R/R, relapsed/refractory.

References

1. Nakajima H, Sato B, Fujita T, Takase S, Terano H, Okuhara M. 1996. New antitumor substances, FR901463, FR901464 and FR901465. I. Taxonomy, fermentation, isolation, physico-chemical properties and biological activities. *J Antibiot (Tokyo)* 49:1196-203
2. Nakajima H, Hori Y, Terano H, Okuhara M, Manda T, et al. 1996. New antitumor substances, FR901463, FR901464 and FR901465. II. Activities against experimental tumors in mice and mechanism of action. *J Antibiot (Tokyo)* 49:1204-11
3. Kaida D, Motoyoshi H, Tashiro E, Nojima T, Hagiwara M, et al. 2007. Spliceostatin A targets SF3b and inhibits both splicing and nuclear retention of pre-mRNA. *Nat Chem Biol* 3:576-83
4. Gao Y, Koide K. 2013. Chemical perturbation of Mcl-1 pre-mRNA splicing to induce apoptosis in cancer cells. *ACS Chem Biol* 8:895-900
5. Fan L, Lagisetti C, Edwards CC, Webb TR, Potter PM. 2011. Sudemycins, novel small molecule analogues of FR901464, induce alternative gene splicing. *ACS Chem Biol* 6:582-9
6. Lagisetti C, Palacios G, Goronga T, Freeman B, Caufield W, Webb TR. 2013. Optimization of antitumor modulators of pre-mRNA splicing. *J Med Chem* 56:10033-44
7. Sakai Y, Yoshida T, Ochiai K, Uosaki Y, Saitoh Y, et al. 2002. GEX1 compounds, novel antitumor antibiotics related to herboxidiene, produced by Streptomyces sp. I. Taxonomy, production, isolation, physicochemical properties and biological activities. *J Antibiot (Tokyo)* 55:855-62
8. Lagisetti C, Yermolina MV, Sharma LK, Palacios G, Prigaro BJ, Webb TR. 2014. Pre-mRNA splicing-modulatory pharmacophores: the total synthesis of herboxidiene, a pladienolide-herboxidiene hybrid analog and related derivatives. *ACS Chem Biol* 9:643-8
9. Sakai T, Asai N, Okuda A, Kawamura N, Mizui Y. 2004. Pladienolides, new substances from culture of Streptomyces platensis Mer-11107. II. Physico-chemical properties and structure elucidation. *J Antibiot (Tokyo)* 57:180-7
10. Mizui Y, Sakai T, Iwata M, Uenaka T, Okamoto K, et al. 2004. Pladienolides, new substances from culture of Streptomyces platensis Mer-11107. III. In vitro and in vivo antitumor activities. *J Antibiot (Tokyo)* 57:188-96
11. Hong DS, Kurzrock R, Naing A, Wheler JJ, Falchook GS, et al. 2014. A phase I, open-label, single-arm, dose-escalation study of E7107, a precursor messenger ribonucleic acid (pre-mRNA) splicesome inhibitor administered intravenously on days 1 and 8 every 21 days to patients with solid tumors. *Invest New Drugs* 32:436-44

12. Eskens FA, Ramos FJ, Burger H, O'Brien JP, Piera A, et al. 2013. Phase I pharmacokinetic and pharmacodynamic study of the first-in-class spliceosome inhibitor E7107 in patients with advanced solid tumors. *Clin Cancer Res* 19:6296-304
13. Seiler M, Yoshimi A, Darman R, Chan B, Keaney G, et al. 2018. H3B-8800, an orally available small-molecule splicing modulator, induces lethality in spliceosome-mutant cancers. *Nat Med* 24:497-504
14. Steensma DP, Wermke M, Klimek VM, Greenberg PL, Font P, et al. 2021. Phase I First-in-Human Dose Escalation Study of the oral SF3B1 modulator H3B-8800 in myeloid neoplasms. *Leukemia* 35:3542-50
15. Scott KA, Kojima H, Ropek N, Warren CD, Zhang TL, et al. 2025. Covalent targeting of splicing in T cells. *Cell Chem Biol* 32:201-18.e17
16. Lazear MR, Remsberg JR, Jaeger MG, Rothamel K, Her HL, et al. 2023. Proteomic discovery of chemical probes that perturb protein complexes in human cells. *Mol Cell* 83:1725-42.e12
17. Assi R, Kantarjian HM, Kadia TM, Pemmaraju N, Jabbour E, et al. 2018. Final results of a phase 2, open-label study of indisulam, idarubicin, and cytarabine in patients with relapsed or refractory acute myeloid leukemia and high-risk myelodysplastic syndrome. *Cancer* 124:2758-65
18. Kirkwood JM, Gonzalez R, Reintgen D, Clingan PR, McWilliams RR, et al. 2011. A phase 2 study of tasisulam sodium (LY573636 sodium) as second-line treatment for patients with unresectable or metastatic melanoma. *Cancer* 117:4732-9
19. Bewersdorf JP, Stahl MF, Taylor J, Chandhok NS, Watts J, et al. 2022. A Phase II Clinical Trial of E7820 for Patients with Relapsed/Refractory Myeloid Malignancies with Mutations in Splicing Factor Genes. *Blood* 140:9065-7
20. Miller VA, Rigas JR, Tong WP, Reid JR, Pisters KM, et al. 1997. Phase II trial of chloroquinoxaline sulfonamide (CQS) in patients with stage III and IV non-small-cell lung cancer. *Cancer Chemother Pharmacol* 40:415-8
21. Jensen-Pergakes K, Tatlock J, Maegley KA, McAlpine IJ, McTigue M, et al. 2022. SAM-Competitive PRMT5 Inhibitor PF-06939999 Demonstrates Antitumor Activity in Splicing Dysregulated NSCLC with Decreased Liability of Drug Resistance. *Mol Cancer Ther* 21:3-15
22. Rodon J, Rodriguez E, Maitland ML, Tsai FY, Socinski MA, et al. 2024. A phase I study to evaluate the safety, pharmacokinetics, and pharmacodynamics of PF-06939999 (PRMT5 inhibitor) in patients with selected advanced or metastatic tumors with high incidence of splicing factor gene mutations. *ESMO Open* 9:102961
23. Vieito M, Moreno V, Spreafico A, Brana I, Wang JS, et al. 2023. Phase 1 Study of JNJ-64619178, a Protein Arginine Methyltransferase 5 Inhibitor, in Advanced Solid Tumors. *Clin Cancer Res* 29:3592-602
24. Bewersdorf JP, Mi X, Lu B, Kuykendall A, Sallman D, et al. 2025. Phase Ib study of PRT543, an oral protein arginine methyltransferase 5 (PRMT5) inhibitor, in patients with advanced splicing factor-mutant myeloid malignancies. *Leukemia* 39:765-9
25. Watts J, Minden MD, Bachiashvili K, Brunner AM, Abedin S, et al. 2024. Phase I/II study of the clinical activity and safety of GSK3326595 in patients with myeloid neoplasms. *Ther Adv Hematol* 15:20406207241275376
26. Cottrell KM, Briggs KJ, Whittington DA, Jahic H, Ali JA, et al. 2024. Discovery of TNG908: A Selective, Brain Penetrant, MTA-Cooperative PRMT5 Inhibitor That Is Synthetically Lethal with MTAP-Deleted Cancers. *J Med Chem* 67:6064-80
27. Briggs KJ, Cottrell KM, Tonini MR, Tsai A, Zhang M, et al. 2025. TNG908 is a brain-penetrant, MTA-cooperative PRMT5 inhibitor developed for the treatment of MTAP-deleted cancers. *Transl Oncol* 52:102264

Downloaded from www.annualreviews.org. Guest (guest) IP: 74.254.78.132 On: Mon, 08 Jun 2026 14:08:54

28. Engstrom LD, Aranda R, Waters L, Moya K, Bowcut V, et al. 2023. MRTX1719 Is an MTA-Cooperative PRMT5 Inhibitor That Exhibits Synthetic Lethality in Preclinical Models and Patients with MTAP-Deleted Cancer. *Cancer Discov* 13:2412-31
29. Belmontes B, Slemmons KK, Su C, Liu S, Policheni AN, et al. 2025. AMG 193, a Clinical Stage MTA-Cooperative PRMT5 Inhibitor, Drives Antitumor Activity Preclinically and in Patients with MTAP-Deleted Cancers. *Cancer Discov* 15:139-61
30. Muraki M, Ohkawara B, Hosoya T, Onogi H, Koizumi J, et al. 2004. Manipulation of alternative splicing by a newly developed inhibitor of Clks. *J Biol Chem* 279:24246-54
31. Iwai K, Yaguchi M, Nishimura K, Yamamoto Y, Tamura T, et al. 2018. Anti-tumor efficacy of a novel CLK inhibitor via targeting RNA splicing and MYC-dependent vulnerability. *EMBO Mol Med* 10
32. Shkreta L, Toutant J, Delannoy A, Durantel D, Salvetti A, et al. 2024. The anticancer potential of the CLK kinases inhibitors 1C8 and GPS167 revealed by their impact on the epithelial-mesenchymal transition and the antiviral immune response. *Oncotarget* 15:313-25
33. Shimizu T, Yonemori K, Koyama T, Katsuya Y, Sato J, et al. 2022. A first-in-human phase I study of CTX-712 in patients with advanced, relapsed or refractory malignant tumors. *Journal of Clinical Oncology* 40:3080-
34. Al-Kali A, Saliba A, Arana Yi CY, Foran JM, Tanoue Y, et al. 2023. Phase 1/2 Multicenter, Open-Label Study of CTX-712 in Patients with Relapsed/Refractory Acute Myeloid Leukemia and Higher Risk Myelodysplastic Syndromes. *Blood* 142:3249-
35. Cui JJ, Jiang P, Deng W, Zhai D, Ling N, et al. 2024. Abstract 5944: Discovery of BH-30236: A novel macrocyclic CLK inhibitor targeting alternative splicing in cancers. *Cancer Research* 84:5944-
36. Burguera AdlF, Cerchione C, Scholl S, Middeke JM, Nitika N, et al. 2024. A phase 1 single-arm, open-label study of emavusertib (CA-4948) in combination with azacitidine and venetoclax in patients (pts) with acute myeloid leukemia (AML) in complete response (CR) with measurable residual disease (MRD). *Journal of Clinical Oncology* 42:TPS6587-TPS
37. Tam BY, Chiu K, Chung H, Bossard C, Nguyen JD, et al. 2020. The CLK inhibitor SM08502 induces anti-tumor activity and reduces Wnt pathway gene expression in gastrointestinal cancer models. *Cancer Lett* 473:186-97
38. Tolcher A, Babiker HM, Chung V, Kim E, Moser J, et al. 2021. Abstract CT112: Initial results from a Phase 1 trial of a first-in-class pan-CDC-like kinase inhibitor (SM08502) with proof of mechanism in subjects with advanced solid tumors. *Cancer Research* 81:CT112-CT
39. Cai G, Bao Y, Li Q, Hsu PH, Xia J, Ngo JCK. 2024. Design of a covalent protein-protein interaction inhibitor of SRPKs to suppress angiogenesis and invasion of cancer cells. *Commun Chem* 7:144
40. He C, Liu B, Wang HY, Wu L, Zhao G, et al. 2022. Inhibition of SRPK1, a key splicing regulator, exhibits antitumor and chemotherapeutic-sensitizing effects on extranodal NK/T-cell lymphoma cells. *BMC Cancer* 22:1100
41. Araki S, Dairiki R, Nakayama Y, Murai A, Miyashita R, et al. 2015. Inhibitors of CLK protein kinases suppress cell growth and induce apoptosis by modulating pre-mRNA splicing. *PLoS One* 10:e0116929
42. Kobayashi A, Clément MJ, Craveur P, El Hage K, Salone JM, et al. 2022. Identification of a small molecule splicing inhibitor targeting UHM domains. *Febs j* 289:682-98
43. Chatrikhi R, Feeney CF, Pulvino MJ, Alachouzos G, MacRae AJ, et al. 2021. A synthetic small molecule stalls pre-mRNA splicing by promoting an early-stage U2AF2-RNA complex. *Cell Chem Biol* 28:1145-57.e6

Downloaded from www.annualreviews.org. Guest (guest) IP: 74.254.78.132 On: Mon, 08 Jun 2026 14:08:54

44. Costales MG, Hoch DG, Abegg D, Childs-Disney JL, Velagapudi SP, et al. 2019. A Designed Small Molecule Inhibitor of a Non-Coding RNA Sensitizes HER2 Negative Cancers to Herceptin. *J Am Chem Soc* 141:2960-74
45. Childs-Disney JL, Yang X, Gibaut QMR, Tong Y, Batey RT, Disney MD. 2022. Targeting RNA structures with small molecules. *Nat Rev Drug Discov* 21:736-62
46. Levin-Furtney S, Thomas M, Harney AM, Shan M, Ivliev A, et al. 2024. REM-422, a Small Molecule MYB mRNA Degrader, Demonstrates Anti-Leukemic Activity As Monotherapy and in Combination with Standards of Care in Preclinical Models of AML. *Blood* 144:826-

Downloaded from www.annualreviews.org. Guest (guest) IP: 74.254.78.132 On: Mon, 08 Jun 2026 14:08:54
